# Lean Enterprise Transformation in VA: a national evaluation framework and study protocol

**DOI:** 10.1186/s12913-019-3919-2

**Published:** 2019-02-04

**Authors:** Anita A. Vashi, Barbara Lerner, Tracy H. Urech, Steven M. Asch, Martin P. Charns

**Affiliations:** 1 Center for Innovation to Implementation, VA Palo Alto Health Care System, 3801 Miranda Avenue, Palo Alto, CA 94304 USA; 2 Department of Emergency Medicine, Stanford University, Stanford, CA 94305 USA; 3 Center for Healthcare Organization and Implementation Research, VA Boston Health Care System, 150 S Huntington Ave, Boston, MA 02130 USA; 4 Division of Primary Care and Population Health, Stanford University, 1265 Welch Rd, Stanford, CA 94305 USA; 5 Department of Health Law, Policy and Management, Boston University School of Public Health, Boston, MA 02118 USA

**Keywords:** Lean, Lean Enterprise transformation, Implementation, Evaluation, Mixed-methods, Veterans health administration

## Abstract

**Background:**

The goal of Lean Enterprise Transformation (LET) is to go beyond simply using Lean tools and instead embed Lean principles and practices in the system so that it becomes a fundamental, collective mindset of the entire enterprise. The Veterans Engineering Resource Center (VERC) launched the Veterans Affairs (VA) LET pilot program to improve quality, safety, and the Veteran’s experience. A national evaluation will examine the pilot program sites’ implementation processes, outcomes and impacts, and abilities to improve LET adoption and sustainment. This paper describes the evaluation design for the VA LET national evaluation and describes development of a conceptual framework to evaluate LET specifically in healthcare settings.

**Methods:**

A targeted literature review of Lean evaluation frameworks was performed to inform the development of the conceptual framework. Key domains were identified by a multidisciplinary expert group and then validated with key stakeholders. The national evaluation design will examine LET implementation using qualitative, survey, and quantitative methods at ten VA facilities. Qualitative data include site visits, interviews, and field observation notes. Survey data include an employee engagement survey to be administered to front-line staff at all pilot sites. Quantitative data include site-level quality improvement metrics collected by the Veterans Services Support Center. Qualitative, quantitative, and mixed-methods analyses will be conducted to examine implementation of LET strategic initiatives and variations in implementation success across sites.

**Discussion:**

This national evaluation of a large-scale LET implementation effort will provide insights helpful to other systems interested in embarking on a Lean journey. Additionally, we created a multi-faceted conceptual framework to capture the specific features of a Lean healthcare organization. This framework will guide this evaluation and may be useful as an assessment tool for other organizations interested in implementing Lean principles at an enterprise level.

**Electronic supplementary material:**

The online version of this article (10.1186/s12913-019-3919-2) contains supplementary material, which is available to authorized users.

## Background

Finding ways to improve quality and efficiency while controlling costs is crucial in American health care. Lean is one promising management approach to achieving these aims. “Lean thinking,” a quality improvement philosophy, is a bundle of concepts, methods and tools originated by the Toyota Motor Company and has been successfully employed in a variety of manufacturing industries [1]. Because of these successes, the healthcare sector has been trying to implement Lean in settings including laboratories [2, 3], intensive care units [4], emergency departments [5, 6], operating rooms [7], and pharmacies [8, 9]; and a few even have tried to implement Lean hospital-wide [10].

Most Lean implementation efforts in healthcare organizations result in modestly positive short-term improvements in targeted areas and have not successfully created transformational change that encompasses the organization’s culture and administrative structures [11–14]. Recognizing that organization-wide change is difficult and requires going beyond the application of Lean tools to foster a culture of continuous learning and improvement, organizations are increasingly turning to Lean Enterprise Transformation (LET) [15, 16]. The goal of LET is to embed Lean principles, strategies, practices and behaviors in the system so that once there is a critical mass of Lean behavior in the enterprise, it becomes a “way of life” and a fundamental, collective mindset of the entire enterprise.

In 2014, the Veterans Health Administration (VHA) faced a crisis following an Office of the Inspector General report describing inappropriate scheduling practices by staff, Veterans experiencing significant delays in receiving care, and alleged deaths related to the delays [17]. The subsequent rebuilding of the Department of Veterans Affairs (VA) healthcare system as a high-performance network included implementing Lean management strategies to improve quality, safety, and the Veteran’s experience [18, 19]. Initial deployment of Lean management at individual VA medical centers began in 2011, however, in 2015, the VHA National Leadership Council formally adopted Lean management as a national quality improvement strategy [20]. This VA LET program consists of a centralized deployment strategy that includes sensei (coaching) services for each pilot site along with programmatic and implementation support and guidance. To our knowledge, this innovative program is the largest, multi-site Lean enterprise transformation effort to date.

We designed an evaluation of the VA LET pilot program that uses principles based in the community-based participatory research (CBPR) paradigm which recommends involvement of stakeholders in research tasks and procedures [21]. This partnership between researchers and operational partners facilitates the integration of knowledge and produces findings that have “real world” value [22]. While CBPR represents our approach to research, we needed a comprehensive conceptual framework to guide our evaluation design. Previously developed conceptual frameworks do not focus on the organizational transformation process or include other concepts unique to Lean, such as respect for people, that would be critical for studying effective LET implementation. The conceptual framework described in this evaluation protocol provides a standardized approach to evaluating the VA LET pilot sites. Additionally, we propose a mixed-methods evaluation design that seeks to: (1) assess the implementation of the strategic initiatives, and variations in implementation success across sites, in order to inform successful implementation of future initiatives; (2) understand the specific contextual characteristics and deployment mechanisms that facilitate success of LET programs; (3) identify provider and staff-level barriers and facilitators to adoption of LET (e.g., knowledge, attitudes, experience); and (4) to determine if and how LET programs result in the transformation of key aspects of an organization, such as culture.

## Methods/design

### Setting

The VHA, comprised of 170 hospitals and 1063 outpatient sites of care, provides inpatient, outpatient, mental health, rehabilitation, and long-term care services to more than nine million enrolled Veterans. VHA, the nation’s largest integrated healthcare system, employs more than 306,000 full time health care professionals and support staff [23].

**I**n 2011 the VA Veterans Engineering Resource Center (VERC) developed the VA LET program and deployment plan that consists of several components considered critical to the successful implementation of Lean. These include conducting annual Transformational Plan of Care (TPOC) events, developing educational and communication plans, identifying and developing value stream areas (VSAs), engaging in continuous daily improvement activities, and receiving expert-level “sensei” facilitation support at the executive and mid-organizational levels [24]. The VERC, the VA Quality Enhancement Research Initiative (QUERI), and investigators at the VA Palo Alto and Boston Healthcare Systems, formed the LET Partnered Evaluation Center to assess the pilot program sites’ implementation processes, outcomes and impacts and abilities to improve LET adoption and sustainment.

This partnered evaluation includes ten VA medical centers that as early adopters of Lean contracted with VERC to deploy the LET framework and receive sensei facilitation. These pilot sites are geographically dispersed across the US and range in VA medical center complexity designation from least (level 3c) to most complex (level 1a). This rating, obtained from VA administrative data sources, is comprised of factors including patient population characteristics, clinical services complexity, research funding, and level of medical training provided (Table 1).

**Table 1:** Characteristics of VA medical centers participating in the LET evaluation

VA facility	LET deployment date	Value stream areas	US census region	FY17 VA hospital complexity rating^b^
Site A	Feb 2011	Inpatient Medicine^a^	Midwest	1a
		Logistics^a^		
		Surgery		
		Primary Care		
		Mental Health		
		Outpatient Medical/Specialty Care		
		Operational Efficiency and Revenue		
Site B	Jul 2012	Inpatient Medicine^a^	West	1a
		Human Resources^a^		
		Emergency Department		
Site C	Jul 2012	Onboarding new residents^a^	South	1b
		Primary Care – hypertension, diabetes^a^		
		Women’s Health – cancer screening^a^		
		Outpatient Specialty Care – Time to consult		
Site D	Jan 2013	Inpatient Medicine^a^	Midwest	1c
		Administrative Efficiency^a^		
		Outpatient Care		
		Access		
Site E	May 2013	Inpatient Medicine^a^	Northeast	1c
		Emergency Department^a^		
		Surgery		
Site F	Sep 2013	Emergency Department (flow)^a^	Midwest	1b
		Hiring process^a^		
		Open scheduling without consults		
		Urology		
Site G	Dec 2014	Primary Care^a^	Northeast	3
		Human Resources		
		Ethical Leadership Culture		
Site H	May 2015	Primary Care^a^	West	1a
		Lifecycle of Workforce Development^a^		
		Mental Health		
Site I	Jun 2015	Access^a^	West	1b
		Veteran Experience^a^		
		Community Care^a^		
		Clinical Care – Ophthalmology, Podiatry, and Audiology^a^		
Site J	Jun 2015	Inpatient flow^a^	West	1a
		Human Resources - Recruitment^a^		
		Human Resources - Retention		

*FY* fiscal year; *LET* Lean Enterprise Transformation, *VA* Department of Veterans Affairs

^a^Indicates the clinical and non-clinical value stream areas evaluated during site visits and follow-up telephone interviews

^b^The Facility Complexity Model is a data driven model that relies on data from VHA corporate databases along with information from VA central office program offices to identify workload and programs (i.e., teaching, research, and complex clinical programs such as cardiac surgery and neurosurgery) at each facility for the purposes of comparing facility complexity. Facilities are categorized into one of five groups: 1a (most complex), 1b, 1c, 2, and 3 (least complex). 1a facilities (highest complexity) are those with high volume, high risk patients, most complex clinical programs, and large research and teaching programs

The VERC instructed each site to select one clinical and one non-clinical value stream for initial focus from the following designated priority areas: inpatient medicine, inpatient surgery, emergency medicine, primary care, outpatient specialty care and outpatient mental health, as well as logistics and human resources.

### Conceptual framework design

The conceptual framework was developed in two stages: an information gathering process and an interactive process. First, to identify key domain areas, we reviewed the literature for existing frameworks and conceptual models that evaluated Lean in health care and focused on successful implementation and organizational transformation. This included searching keywords including Lean, implementation, model and/or framework, and “snowballing” based on sentinel papers that emerged (including both peer-reviewed literature and grey literature such as government reports). Additional domain areas were solicited from key informants that included our partners involved in implementing Lean and partners responsible for funding the Lean program.

Next, literature review results and key informant input were evaluated using an informed, interactive process. We convened a multidisciplinary academic research group (*n* = 12) with expertise in Lean, quality improvement, organizational theory, management, and program evaluation. Initial key domains and constructs were selected during a half-day in-person meeting, which was then followed by a series of biweekly virtual meetings for refinement. Agreement on relevant domains and constructs was reached through discussion and consensus. The final model was presented and discussed with key stakeholders, including healthcare providers, VHA policymakers and national experts outside of the VHA for input and modification.

### Evaluation design

We propose a three-part mixed-methods design to evaluate LET implementation at the pilot sites. Interview teams will first conduct semi-structured interviews and site visits; findings will be qualitatively analyzed to identify themes and patterns of successful LET implementation. The results will also be used to develop a Lean implementation survey, the second component of the evaluation. The third component includes an analysis of relevant quality improvement metrics created and collected by the VHA to assess changes in clinical and non-clinical processes and outcomes at each VA facility. Below, we present the details of each design component.

### Recruitment and sampling

#### Qualitative Site Visit/Interviews

The pilot sites will be invited to participate in the evaluation per an introductory email from the VERC leadership and the QUERI director. For each site, we will identify a site contact to assist with identifying, inviting, and scheduling the key informant interviews and coordinating the site visit activities. Prior to any site visit, the site contact will provide information about the site’s history with process improvement and the LET program (i.e., when they first started working with VERC to implement LET, current and planned value streams, date of last TPOC event, etc.). Interview participants will include the medical center director and other executive leadership (e.g., Chief of Staff), the director of systems redesign/process improvement, the Lean senseis (coaches), the two value stream process owners - who will typically be the Director/Service Chief of the departments in which the value stream will occur (e.g., Inpatient Medicine, Human Resources), middle managers and front-line supervisors working on one value stream, and front-line staff working on the other value stream.

#### Staff survey

All ten pilot sites will be invited to participate in a web-based survey. With approval from the medical center director, the survey will be administered to all employees below the level of executive leadership. Following the Dillman procedure [25], the medical center director will send to all staff, at one week intervals, a series of email notifications: a pre-survey email informational notice, an email containing the link to the online survey, and two follow-up reminder emails.

#### Quantitative Analysis

The VHA has a comprehensive database of metrics to support performance improvement. One such tool is a sophisticated matrix of over 25 measures and measure sets known as Strategic Analytics for Improvement and Learning (SAIL) [26, 27]. It includes all Veteran-relevant metrics reported to the Centers for Medicare and Medicaid Services and the National Committee for Quality Assurance, as well as assessments of factors thought to enable quality and safety improvement (e.g., employee morale, nursing turnover, leadership vacancies, and selected utilization metrics). The SAIL metrics related to the targeted value stream areas will be the focus of the quantitative stage of the evaluation.

### Data collection

#### Qualitative Site Visit/Interviews

Three rounds of interviews will be conducted at approximately six-month intervals. The first round will serve as a baseline assessment of the sites’ Lean status. They will be conducted during a two-day on-site visit by at least two evaluation members and the subsequent rounds will be telephone-based interviews. During the in-person visit, the site-visit team will tour the facility, paying special attention to visual management tools such as huddle boards, recognition for Lean participation and accomplishments, and other visual displays related to Lean efforts. They will note whether the visual displays contain relevant and up-to-date measures, and reflect alignment between the individual work area’s focus of efforts and the medical center’s strategic goals. The visit will also include attending huddle meetings and project report-out meetings. The site-visit team will document their observations using a Field Observations and Impressions Summary form (Additional file 1). Using the structured form, the team will describe their observations as they relate to framework domain areas and summarize their overall impressions of the site. Photographs of the visual management displays will also be collected. For consistency and to evaluate change over time, the Round 2 interviews will target the same individuals in the Round 1 value stream areas (when applicable) and at least one member of the Round 1 site-visit team will conduct Round 2 interviews. Round 3 interviews will focus on obtaining a summative assessment from three to five key informants (two executive leaders, two value stream process owners and the system redesign director) regarding the site’s Lean journey to date.

Interview guide questions, based on our conceptual framework’s domains and constructs, will elicit key informants’ current perceptions and knowledge about the LET deployment efforts at their facility. Guide development will be iterative and reviewed by Lean experts and operational partners to allow for input and modification. The guides will include questions specific to the interview round as well as questions revisited each time to document progress in the site’s Lean journey. For example, only Round 1 interviews will include questions to assess the site’s history with process improvement and to better understand the cultural context of the site. The Round 2 interview guide will incorporate questions about the effects of management changes and ongoing commitment to Lean. The final interview round (Round 3) will assess the trajectory of Lean efforts, especially to determine whether prior accomplishments have been sustained, whether Lean has been spread to additional parts of the organization, and the lessons learned. Unique interview guides will be created for each interview type (leadership, value stream owners, middle managers, front-line staff, senseis, and system redesign). All interviews will be conducted using the interview guide appropriate for the individual or group (middle manager and front-line staff) being interviewed. Trained members of the evaluation team will conduct the interviews designed to take approximately one hour to conduct. The interviews will be audiotaped and subsequently transcribed verbatim with the participant’s permission.

A codebook will be developed using a deductive open-coding approach with codes largely generated from the conceptual framework [28]. Trained coders will code interview transcripts and site visit field notes in Atlas.ti. Coders will meet weekly, and with the larger evaluation team bi-weekly, to ensure a shared interpretation of the codes and to discuss potential inductive generated codes. Agreement between coders will be monitored by quarterly Kappa testing, with a target kappa coefficient of 0.80. Secondary coding will occur for 30% of all interviews for quality assurance. Differences will be resolved by consensus.

#### Staff survey

The survey items will be generated to operationalize each domain from the conceptual framework. Items will be written as close-ended, declarative statements from the staff member’s perspective, describing staff behaviors, feelings, perceptions and beliefs about Lean implementation at their site, as well as their observations of their own manager’s behavior. Next, a panel consisting of Lean content experts, front-line staff, and a survey development expert will review the initial item pool and rate each item for validity and clarity to create a content validity index (CVI) assessing fit of each item to its conceptual domain [29]. Items having a CVI of 0.7 or greater will be retained. Other survey items will be eliminated or modified based on the expert panel review. The resulting survey will be piloted by administering it to staff at eight VHA medical centers that are not a part of the evaluation but are involved in process improvement efforts. Using the pilot data, we will conduct a serially exploratory factor analysis with at least three items per factor, and reliable fit statistics [30]. We will then examine these items using a confirmatory factor analysis, looking for appropriate fit statistics, loadings, and clinical relevance [31, 32].

#### Quantitative Analysis

We will collect SAIL data from 2010 to 2017 from the Veterans Services Support Center, which provides informatics tools, data reports, and analytics services to support improvements in Veterans’ healthcare [33]. The SAIL measures organized by the targeted value stream areas are listed in Additional file 2*.*

### Data analysis

#### Qualitative Site visit/Interviews

Following Rounds 1 and 2 interviews, the sites transcripts will be coded and each site will be scored on the conceptual framework constructs using a 0–4 scale to indicate the extent to which each construct exists at the site at the time of the interviews (Table 2). This method of coding and scoring is based on a rating system used previously to evaluate organizational transformation [34, 35]. An extensive training process will ensure all team members have consistent understanding of the construct definition and examples.

**Table 2:** Rating scale to score the Lean transformation constructs

Scale	Evidence level	Rating description
0	No evidence of existence	There is little to no evidence that the construct is present at the site level, appears to have neutral effect (purely descriptive) or is only mentioned generically without evidence of positive or negative influence.
		• No interviewee describes this construct as existing at this time.
		• The construct is mentioned only in passing or at a high level without examples or evidence of actual, concrete descriptions of how that construct manifests.
		• There is insufficient information to make an inference about the generally positive or negative influence.
1	Limited evidence of existence	The construct is only inconsistently present as a positive influence at this site. Interviewees make general statements about the construct manifesting in a positive way but without several different examples.
		• One interviewee describes explicit examples of how this construct manifests itself in a positive way.
		• Other interviewees make general statements about the construct manifesting in a positive way but without concrete examples. There may be reports of mixed effects of the construct on implementation but with a general overall positive.
		• There is little information to indicate that this construct affects all areas of both value streams or multiple groups/units/departments within the site.
		• The interviewee is talking about wanting to do it, but it is not yet being implemented. At least they are aware of the issue.
2	Some of the time evident	The construct is stable but narrowly present as a positive influence within some areas or at a few levels of the site.
		• Two or more interviewees similarly describe this construct and how it is manifested. Are they on the same page or not?
		• Two or more interviewees describe explicit examples of how this construct manifests itself in a positive way.
		• Other interviewees make statements about the construct manifesting in a positive way with some concrete examples. There may be a few conflicting reports indicating mixed effects of the construct on implementation but most show an overall positive impact.
		• There is some information to indicate that this construct affects all areas of both value streams or multiple groups/units/departments within the site.
3	Much of the time evident	This construct is stable and present within many areas or at many levels of the site but there are still some pockets of the organization in which this construct is lacking.
		• Two or more interviewees from different organizational groups (leaders vs middle managers vs frontline) similarly describe this construct and how it is manifested. Are they on the same page or not?
		• The majority of interviewees describe explicit examples of how this construct manifests itself in a positive way.
		• Other interviewees make concrete statements about the construct manifesting in a positive way with various types of examples. There may be a few conflicting reports indicating mixed effects of the construct on implementation but in general there is a clear overall positive impact.
		• There is a lot of information to indicate that this construct affects all areas of both value streams or multiple groups/units/departments within the site.
4	Evidence exists firmly embedded in the organiza-tion	The construct is stable and broadly established at the site level.
		• Nearly all interviewees from different organizational groups (leaders vs middle managers vs frontline) similarly describe this construct and how it is manifested. Are they on the same page or not?
		• The majority of interviewees describe explicit examples of how this construct manifests itself in a positive way.
		• Other interviewees make concrete statements about the construct manifesting in a positive way with many examples. There may be a few conflicting reports indicating possible mixed effects of the construct on implementation but there is virtually a clear overall positive impact.
		• There is overwhelming information to indicate that this construct affects virtually all areas of both value streams or multiple groups/units/departments within the site.
Miss	Missing	Interviewee(s) were not asked about the presence or influence of the construct. Interviewee(s) lack of knowledge about a construct does not necessarily indicate missing data and may instead indicate the absence of the construct.

Members from the evaluation interview teams will be paired and assigned a set of constructs to rate for all the sites. This will ensure consistent rating scores across all sites and timeframes. For each construct, the raters will be provided all coded narrative passages related to their assigned constructs from the transcripts. Raters will independently review the evidence and rate their assigned constructs. Next, the pairs will meet to review their scores and if there is a discrepancy they will review the data to reach a consensus score. Raters may indicate if there is insufficient data provided from the narrative passages to reliably rate the construct. Raters will also summarize their assessments to support their rating and note exemplar quotes that best illustrate the construct and associated rating. After construct ratings are complete, construct scores will be summed to create an overall score for each site, for each round. Difference and percent change from Round 1 to Round 2 for each construct will be calculated.

The rating scores will be used to compare each site’s progress and degree of Lean implementation compared to the other pilot sites. Based on the total summed rating score, sites will be grouped into high, medium and low Lean maturity sites. Lean maturity describes the stage in an organization’s Lean journey. This ordering will facilitate identification of differences between the groups, providing insight into facilitators and barriers to successful implementation. We will use Round 3 interview data to generate summative assessments of the sites’ Lean journey thus far and to compile a list the significant lessons learned by the leaders of the LET implementation process.

#### Staff Survey

The survey data will be analyzed to compare sites on staff engagement and Lean spread within sites. We will test whether the survey measures are associated with the sites’ level of implementation as measured by the qualitative data.

First, a confirmatory factor analysis will be conducted and internal scale reliabilities will be examined. We will then examine basic descriptive statistics, associations among different measures at the individual respondent level, and differences in scales and key individual items associated with different respondent demographics (such as profession/occupation, level of supervisory authority, gender and tenure). Because individual staff can be associated with the value stream area that they have worked on we can assess the distribution and extent of staff involvement in Lean, and whether staff that have participated in a particular value stream report more use of Lean tools and techniques and are more engaged in their medical center than those working in other value streams or those who have not been involved in value stream Lean efforts. In addition, we will test whether involvement in a value stream that is a designated priority for a given medical center is even more strongly associated with the survey measures. We will also assess whether participation in continuous daily improvement activities separate from value stream involvement is associated with increased engagement. Finally, we will analyze scales and key individual items using random effects models, where employee demographic characteristics are entered as fixed effects and value streams and medical centers as random effects.

#### Quantitative Analysis

Sites will be longitudinally evaluated and compared on improvements in SAIL measures in the respective value streams relevant to the areas they selected for Lean improvement efforts. We will measure the change in site-specific SAIL measures using an interrupted time series design. This is a robust technique to evaluate the immediate and sustained effect of a change in healthcare delivery, such as a policy change or quality improvement intervention. All data captured from 2010 to six months after LET implementation will be used for calculating trends prior to LET implementation (pre-implementation period). This approach allows for a six month implementation period (e.g., washout period). Data from six months after LET implementation through the end of 2017, will be used to calculate trends in SAIL measures after LET implementation (post-implementation period). Wald tests will be used to calculate the significance between pre- and post-SAIL trends. Estimates will be provided for each individual SAIL measure as well as in aggregate for each site. Two-sided *p*-values less than 0.05 will be considered statistically significant. Statistical analyses will be conducted using R [36].

## Discussion

Based on the review of the literature, the evaluation team selected the Organizational Transformation Model (OTM) as a starting point for the development of a conceptual framework model [37]. The OTM is a model for moving organizations from short-term, isolated performance improvements to sustained, reliable, organization-wide transformation of healthcare delivery. The model identifies five elements critical for successful transformation: (1) Impetus to transform; (2) Leadership commitment to quality; (3) Improvement initiatives that actively engage staff in meaningful problem solving; (4) Alignment to achieve consistency of organization goals with resource allocation and actions at all levels of the organization; and (5) Integration to bridge traditional intra-organizational boundaries among departments and staff roles. The OTM is most often used in process improvement environments [12, 34, 38, 39]. To capture the specific features of a Lean organization, we integrated the OTM with concepts derived from our literature review, input from our operational partners that included additional Lean specific concepts such as just culture, use of metrics for decision-making, capability development, and a focus on the customer, in this case, specifically the Veteran.

Our final conceptual model for evaluating Lean transformation in healthcare settings includes ten domains and 24 constructs (Table 3).

**Table 3:** Conceptual framework domains and constructs to evaluate VA’s Lean Enterprise Transformation

Domain No.	Domain	Construct
1	Impetus to transform	• Leadership uses an identified impetus to engage staff in Lean Enterprise Transformation (LET) efforts --Initial impetus --Continued impetus
2	Leadership commitment to quality	• Senior leaders demonstrate a long-term strategy/vision for LET implementation that reflects an understanding of how the LET components interact and build over time.
		• Leadership's commitment to overall LET implementation.
		• Senior leaders engage and encourage middle management (service chiefs, nurse managers, frontline supervisors) to support their staff to participate in LET activities.
		• Senior leaders participate themselves in a variety of Lean activities.
3	Organizational culture	• Problems, mistakes/errors, poor performance on metrics are seen as opportunities for growth, change, and improvement rather than an opportunity to blame (focusing on the system rather than the individual), a just culture.
		• Senior leaders hold middle managers accountable for meeting LET objectives.
		• Respect for People (culture of respect, constructive, respectful conflict, recognizing systems issues, etc) exists.
		• Barriers to quality improvement are systematically identified and resolved.
		• Improved processes and procedures are implemented in other appropriate areas of the organization (i.e., spread.)
4	Informed decision making	• Valid data and information are readily available for key Lean/improvement processes (e.g., TPOC, VSAs, RPIEs).
5	Integration across boundaries	• Staff from different work areas work together effectively and willingly across organizational boundaries on QI or Lean activities such as Value Stream efforts (e.g., engaging other services as needed to accomplish LET objectives).
		• Staff from different disciplines work together effectively and willing across organizational boundaries on QI or Lean activities such as Value Stream efforts (e.g., engaging other services as needed to accomplish LET objectives).
6	Alignment - Alignment of improvement/ Innovation across the organization	• Leadership and managers effectively use incentives and reward structures to encourage involvement in LET efforts; they are not rewarded for maintaining the status quo.
		• Members of leadership and/or service chiefs encourage or support improvement activities provides resources (e.g., protected time, supplies).
7	Alignment - Key initiatives aligned to strategies	• True North metrics/goals were observed.
		• The organization’s Lean activities (e.g., continuous daily improvement and value stream efforts) are aligned with True North metrics/goals.
8	Communication	• Successes are celebrated and broadcasted to generate awareness and enthusiasm.
		• Staff receive communication orally, on web site or physical displays from higher levels of the organization regarding Lean expectations, goals, etc.
		• Staff input regarding Lean activities is received by higher levels of the organization and acknowledged.
9	Capability development	• Medical Center has a clear plan for training staff in Lean principles (which may include formal and on-the-job training).
		• Staff have been trained in Lean principles.
		• Staff who have gone through training are using skills in improvement work.
		• Managers, supervisors and/or consultants such as sensei and systems redesign staff help staff use their newly-learned skills.
10	Veteran/patient engagement	• Voice of the Veteran (i.e., patient/family) is used as part of the improvement process.

*QI* quality improvement, *RPIE* rapid process improvement event, *TPOC* transformational plan of care, *VSA* value stream area

The first domain, Impetus to Transform, requires leadership to identify and continuously use an incident, goal or passion, meaningful to the entire organization, to create a sense of urgency among the staff to participate in the Lean journey. The Leadership Commitment to Quality domain focuses on the abilities of the leaders to create and implement a long-term vision for Lean within the organization and recognize the multi-faceted complexity of the transformational change process. Also included in this domain is the expectation that senior leaders engage middle management to both participate and support their staff in participating in Lean efforts while also actively participating themselves the Lean activities (e.g., they do regular gemba walks, attend huddles, or rapid process improvement workshops.) The Organizational Culture domain incorporates several aspects of culture including the concept of a just culture in which mistakes and errors and poor performance on metrics are seen as opportunities for improvement rather than to blame individuals. Respect for all individuals supports a psychologically safe environment in which staff may experiment and propose innovative ideas without fear of repercussions from management or colleagues. For example, individual’s opinions are sought after and valued by their fellow staff, their managers and the leadership. This domain also acknowledges the need for individual ownership and accountability for their commitment to meeting the organization’s Lean objectives. One of the central requirements of a Lean organization is to develop a culture as a learning organization such that barriers to quality improvement are systematically identified and resolved. The last construct within this domain is that of spread; successfully improved processes and procedures are spread from one area to other appropriate areas of the organization and similarly lessons learned can be shared to increase the rate of a successful transformation. The fourth domain incorporates one of the central tenants of Lean management- making informed decisions based on the results from valid and meaningful metrics. Further, the data should be up to date, readily available and widely available especially to those working on related projects. The Integration across Boundaries domain encompasses the idea of staff from different work areas (clinical and administrative) and disciplines (e.g., nurses and physicians) working together effectively on improvement efforts, which helps to break down silos and build a stronger, more respectful organizational culture. Our conceptual framework includes two domains related to Alignment. These domains incorporate several ideas such as leadership and management should use incentives and reward structures as well as provide resources such as protected time to encourage Lean involvement by staff. Additionally, the organization ensures that selected metrics for value stream areas and continuous daily improvement efforts are in line with the organization’s True North or strategic goals. Communication is another domain that addresses the need for staff to receive regular and consistent information from higher levels of the organization regarding Lean expectations and goals. It is also important to encourage staff input regarding Lean activities and that managers and leaders authentically consider and acknowledge it. Equally valuable is to broadcast and celebrate Lean successes to generate awareness and enthusiasm. The Capability Development domain addresses the need to have and implement a clear educational plan that allows individuals at all levels of the organization to obtain training about Lean concepts and the use of Lean tools. Training can include both traditional classroom time as well as just-in-time training, for example at the start of a value stream project. To enforce training, staff must have the opportunity to use their new skills and receive coaching from supervisors and colleagues already Lean skilled. Patient Engagement is the final domain. The voice of the customer (i.e., patient in the healthcare setting) is the concept of engaging patients in the Lean transformation by getting their input via methods such as surveys and interviews, as well as having them participate on Lean related committees and activities such as rapid process improvement workshops.

This evaluation will use a newly developed conceptual framework and a mixed-methods approach to characterize and describe lessons learned from a widespread effort to implement LET in the VHA. Staff commentary coupled with detailed data analysis will support a more thorough understanding of outcomes and impacts, including cultural change, at the enterprise level—across multiple healthcare facilities implementing LET. This approach will fill an evidence gap critical to healthcare leaders and quality improvement staff within healthcare organizations planning to adopt Lean. The lessons learned by the LET pilot sites may allow more effective implementation of Lean practices and help transform the organizations’ culture to one of ongoing process improvement that benefits the organization, the staff and the patients they serve.

## Additional files

Additional file 1: Field observations and impressions summary form. (DOCX 18 kb)

Additional file 2: VA SAIL measures by value stream area. (DOCX 22 kb)

## Abbreviations

CBPR: Community-based participatory research
CVI: Content validity index
LET: Lean enterprise transformation
OTM: Organizational transformation model
QUERI: Quality enhancement research initiative
SAIL: Strategic analytics for improvement and learning
TPOC: Transformational plan of care
VA: Department of Veterans Affairs
VERC: Veterans engineering resource Center
VHA: Veterans Health Administration
VSA: Value stream area

## References

[1] Stone K (2012). Four decades of lean: a systematic literature review. Int J Lean Six Sigma.

[2] Persoon T, Zaleski S, Frerichs J (2006). Improving preanalytic processes using the principles of lean production (Toyota production system). Am J Clin Path.

[3] Clark DM, Silvester K, Knowles S (2013). Lean management systems: creating a culture of continuous quality improvement. J Clin Pathol.

[4] Shannon R, Frndak D, Grunden N, Lloyd JC, Herbert C, Patel B (2006). Using real-time problem solving to eliminate central line infections. Jt Comm J Qual Patient Saf..

[5] Vashi AA, Haji-Sheikhi F, Nashton LA, Ellman J, Rajagopal P, Asch S. Applying lean principles to reduce wait times in the emergency department. Mil Med. 2018. 10.1093/milmed/usy165. [Epub ahead of print]. 10.1093/milmed/usy165 PMC13715879 30007347

[6] Allaudeen N, Vashi A, Breckenridge JS, Haji-Sheikhi F, Wagner S, Posley KA (2017). Using lean management to reduce emergency department length of stay for medicine admissions. Qual Manag Health Care.

[7] Coffey C, Cho ES, Wei E, Luu A, Ho M, Amaya R (2018). Lean methods to improve operating room elective first case on-time starts in a large, urban, safety net medical center. Am J Surg.

[8] Hammoudeh S, Amireh A, Jaddoua S, Nazer L, Jazairy E (2018). Applying lean management to reduce patient waiting time and improve satisfaction at a hospital outpatient pharmacy. Pharmacotherapy.

[9] John N, Snider H, Edgerton L, Whalin L (2017). Incorporation of lean methodology into pharmacy residency programs. Am J Health-Syst Pharm.

[10] Furman C, Caplan R (2007). Applying the Toyota production system: using a patient safety alert system to reduce error. Jt Comm J Qual Patient Saf.

[11] Kaplan GS, Patterson SH, Ching JM, Blackmore CC (2014). Why lean doesn't work for everyone. BMJ Qual Saf.

[12] Patri R, Suresh M (2018). Factors influencing lean implementation in healthcare organizations: an ISM approach. Int J Healthc Manag.

[13] Moraros J, Lemstra M, Nwankwo C (2016). Lean interventions in healthcare: do they actually work? A systematic literature review. Int J Qual Health Care.

[14] Mazzocato P, Savage C, Brommels M, Aronsson H, Thor J (2010). Lean thinking in healthcare: a realist review of the literature. Qual Safe Health Care.

[15] Nightingale D, Srinivasan J (2011). The seven principles of enterprise transformation. Beyond the lean revolution: achieving successful and sustainable Enterprise transformation.

[16] Koenigsaecker G (2013). Leading the lean enterprise transformation.

[17] VA Office of Inspector General. Veterans Health Administration – review of alleged patient deaths, patient wait times, and scheduling practices at the Phoenix VA Health Care System. 2014. http://www.va.gov/oig/pubs/vaoig-14-02603-267.pdf. Accessed 22 Aug 2018.

[18] Shulkin DJ (2016). Beyond the VA crisis--becoming a high-performance network. N Engl J Med.

[19] Buell R. A transformation is under way at the US veterans affairs. We got an inside look 2016. https://hbr.org/2016/12/a-transformation-is-underway-at-u-s-veterans-affairs-we-got-an-inside-look. Accessed 22 Aug 2018.

[20] Woodward-Hagg H, Hemphill R. Lean Enterprise Transformation within VHA. Forum: Translating research into quality health care for Veterans. 2015. http://www.hsrd.research.va.gov/publications/forum/aug15/default.cfm?ForumMenu=aug15-1. Accessed 22 Aug 2018.

[21] Israel BA, Schulz AJ, Parker EA, Becker AB (1998). Review of community-based research: assessing partnership approaches to improve public health. Annu Rev Public Health.

[22] Cashman SB, Adeky S, Allen AJ, Corbum J, Israel BA, Montano J (2008). The power and the promise: working with communities to analyze data, interpret findings, and get to outcomes. Am J Public Health.

[23] U.S. Department of Veterans Affairs. Veterans health administration. 2018. http://www.va.gov/health/aboutVHA.asp. Accessed 22 Aug 2018.

[24] Hagg, H. Large system transformation within healthcare organizations utilizing lean deployment strategies. Worcester Polytechnic Institute 2013. http://digitalcommons.wpi.edu/etd-dissertations/415. Accessed 20 Sep 2018.

[25] Dillman D (2007). Mail and Internet surveys: the tailored design method.

[26] Veterans Health Administration. Strategic Analytics for Improvement and Learning (SAIL). 2018. http://www.va.gov/QUALITYOFCARE/measure-up/Strategic_Analytics_for_Improvement_and_Learning_SAIL.asp. Accessed 22 Aug 2018.

[27] Rubin R (2018). VA announces plan to improve worst-performing medical centers. JAMA.

[28] Hsieh H-F, Shannon SE (2005). Three approches to qualitative content analysis. Qual Health Res.

[29] Polit DF, Beck CT (2006). The content validity index: are you sure you know what's being reported? Critique and recommendations. Res Nurs Health.

[30] Schmitt T (2011). Current methodological considerations in exploratory and confirmatory factor analysis. J Psychoeduc Assess.

[31] Kim KH (2005). The relation among fit indexes, power, and sample size in structural equation modeling. Struct Equa Modeling.

[32] Jackson DL, Gillaspy JA, Purc-Stephenson R (2009). Reporting practices in confirmatory factor analysis: an overview and some recommendations. Psychol Methods.

[33] Department of Veterans Affairs. VHA Support Service Center Capital Assets (VSSC). http://www.data.va.gov/dataset/vha-support-service-center-capital-assets-vssc. Accessed 22 Aug 2018.

[34] VanDeusen Lukas C, Engle RL, Holmes SK, Parker VA, Petzel RA, Nealon Seibert M (2010). Strengthening organizations to implement evidence-based clinical practices. Health Care Manag Rev.

[35] Babich LP, Charns MP, McIntosh N, Lerner B, Burgress JF, Stolzmann KL (2016). Building systemwide improvement capability : does an organization's strategy for quality improvement matter?. Qual Manag Health Care.

[36] R Core Team. R: a language and environment for statistical computing. R Foundation for statistical computing. 2013. http://www.R-project.org. Accessed 20 Sep 2018.

[37] Lukas CV, Holmes SK, Cohen AB, Restuccia J, Cramer IE, Shwartz M (2007). Transformational change in health care systems: an organizational model. Health Care Manag Rev.

[38] Sullivan JL, Adjognon OL, Engle RL, Shin MH, Afable MK, Rudin W (2018). Identifying and overcoming implementation challenges: experience of 59 noninstitutional long-term services and support pilot programs in the veterans health administration. Health Care Manag Rev.

[39] Shin MH, Rivard PE, Shwartz M, Borzecki A, Yaksic E, Stolzmann K (2018). Tailoring an educational program on the AHRQ patient safety indicators to meet stakeholder needs: lessons learned in the VA. BMC Health Serv Res.

