# Supplementary material for: Lean Enterprise Transformation in VA: a national evaluation framework and study protocol
Source: BMC Health Serv Res. 2019 Feb 4;19:98. doi: 10.1186/s12913-019-3919-2 (PMC6360753; doi:10.1186/s12913-019-3919-2)
Supplement: Supplementary file 1 — Field observations and impressions summary form. (DOCX 18 kb) [file 12913_2019_3919_MOESM1_ESM.docx]

**Additional file 1. Field observations and impressions summary form.**

| **Visual Displays (visual observation)** | | |
| --- | --- | --- |
| **Construct** | **Construct Operationalization Examples** | **Observations (overall assessment of all visual displays)** |
| **Basic Description** | - Type (e.g., MESS, PDSA, 5-S diagram, etc.). - Where located? (e.g., available to public, easy access by staff, hidden away) - Completeness of displays - Up-to-dateness of entries (within past week, past month, older than 1 month) |  |
| **Evidence of Strategic Plan/Medical Center Priorities (“Alignment”)**  (nb: site visitor should reference Medical Center Fact Sheet) | - Examples include written statements, logos, goal statements related to Strategic Plan |  |
| **Evidence of Data Use (“Informed Decision Making”)** | - Data or measures are displayed? - Up-to-date? - Example of displayed measure and use: - Benchmark displayed (from within Medical Center? Within VISN? Within VA?) |  |
| **Evidence of Working with Other Workgroups (Integration)** | - Visual from other workgroups (e.g., names, other workgroup’s data) |  |
| **Overall Impressions**  **And Other Observations** | Positive, negative, neutral, other  observations |  |

| **Meeting Observations** | | |
| --- | --- | --- |
| **Construct** | **Construct Operationalization Examples** | **Observations (overall assessment of all meetings)** |
| **Basic Description** | - Types (huddles at unit, management, or department head level, bed meeting) - Suitability of meeting location - People presenting (leadership, staff, department specific, hospital wide?) - General topics discussed - Up-to-dateness of discussions |  |
| **Evidence of Strategic Plan/Medical Center Priorities (“Alignment”)**  (reference Medical Center Fact Sheet) | • Discussion references strategic plan, key initiatives, goals, priorities  • Who involved in discussion of these priorities? |  |
| **Evidence of Data Use (“Informed Decision Making”)** | - Data or measures are referenced or discussed - Benchmarks discussed - Who involved in discussion of these measures? |  |
| **Evidence of Work Across Workgroups/Units/Departments**  **(“Integration”)** | - Members from different work groups, units, and departments present. Which units? - Discussion involves input from different workgroups. Who? |  |
| **Just Culture/Organizational Culture** | - Language of blame? - System problem vs. individual problem? |  |
| **Individual Accountability** | - People actively take responsibility or ownership for project, its progress etc.?   Who takes responsibility, etc.   - Lean skills demonstrated (e.g., terms used) |  |
| **Leadership** | - Leaders described as involved, supportive - Who described? Who did describing? |  |
| **Overall Impressions**  **And Other Observations** | - Positive, negative, neutral, other |  |

Describe your **overall impression** of the site. In addition, remember to note **any relevant information/data that you observed or discussed during the facility tour with the site contact**.

**(highlights, red flags, other)**

| **Feedback:** |
| --- |
