# Supplementary material for: Lean Enterprise Transformation in VA: a national evaluation framework and study protocol
Source: BMC Health Serv Res. 2019 Feb 4;19:98. doi: 10.1186/s12913-019-3919-2 (PMC6360753; doi:10.1186/s12913-019-3919-2)
Supplement: Supplementary file 2 — VA SAIL measures by value stream area. (DOCX 22 kb) [file 12913_2019_3919_MOESM2_ESM.docx]

**Additional file 2. VA SAIL measures by value stream area.**

| **Value stream area** | **SAIL measure** | **Measure definition*** |
| --- | --- | --- |
| Inpatient Medicine | Preventable in-hospital complications | Observed number of hospitalizations with occurrence of any of seven complications (wound infection, hospital acquired pneumonia, shock or cardiac arrest, upper gastrointestinal bleeding, hospital acquired sepsis, deep vein thrombosis, and central nervous system complications) divided by the expected number of hospitalizations with occurrence of any of the seven complications |
| Inpatient Medicine | Healthcare associated infections | Infections caused by a wide variety of common and unusual bacteria, fungi and viruses while receiving medical care.  Separate measures reported for:   - Central line associated bloodstream infection (CLAB) - Catheter associated urinary tract infection (CAUTI) - Methicillin-Resistant Staphylococcus aureus (MRSA) - Ventilator associated events |
| Inpatient Medicine | Patient safety index (PSI) | Derived from a set of patient safety indicators developed by the Agency for Healthcare Research and Quality (AHRQ) that are widely used to reflect quality of care inside hospitals, as well as across geographic areas, to focus on potentially avoidable complications and iatrogenic events  10 indicators included in SAIL:  • Pressure ulcer (PSI 3)  • Death among surgical inpatients with serious, treatable complications (PSI 4)  • Iatrogenic pneumothorax (PSI 6)  • Central venous catheter-related blood stream infections (PSI 7)  • Perioperative hemorrhage or hematoma (PSI 9)  • Postoperative Acute Kidney Injury Requiring Dialysis (PSI 10)  • Postoperative respiratory failure (PSI 11)  • Perioperative pulmonary embolism or deep vein thrombosis (PSI 12)  • Postoperative sepsis (PSI 13)  • Postoperative wound dehiscence (PSI 14) |
| Inpatient Medicine | Acute care risk adjusted LOS | Calculated by first estimating LOS index, measured as the sum of the actual LOS divided by the sum of the expected LOS for a hospital. The risk adjusted LOS is then calculated by multiplying the LOS index by the grand mean LOS for all VA hospitals. |
| Inpatient Medicine | All cause hospital-wide 30-day readmission rate | The model uses the CMS hospital-wide readmission measure but also includes in the model unplanned readmissions within 30 days of hospital discharge for all conditions among adult patients 18 and older. This measure is presented as a single summary score derived from the results of five models (patient cohorts): Medicine, Surgery/Gynecology, Cardiorespiratory, Cardiovascular, and Neurology. |
| Inpatient Medicine | 30-day readmission rate, Medicine cohort | See definition for “All cause hospital-wide 30-day readmission rate.” |
| Inpatient Medicine | CMS 30-day AMI RSRR | CMS measures AMI RSMR as the ratio of the number of predicted deaths within 30 days of hospital admission in AMI patients to the expected number of deaths within 30-day of hospital admission in AMI patients, multiplied by the national unadjusted 30-day mortality rate. The predicted deaths are adjusted for patient and facility characteristics, while the expected deaths are adjusted for patient characteristics only. |
| Inpatient Medicine | CMS 30-day CHF RSRR | CMS measures CHF RSRR as the ratio of the number of predicted readmissions within 30 days of discharge in CHF patients to the expected number of readmissions within 30-day of hospital discharge in CHF patients, multiplied by the national unadjusted 30-day readmission rate. The predicted readmissions are adjusted for patient and facility characteristics, while the expected readmissions are adjusted for patient characteristics only. |
| Inpatient Medicine | CMS 30-day Pneumonia RSMR | CMS measures Pneumonia RSMR as the ratio of the number of predicted deaths within 30 days of hospital admission in pneumonia patients to the expected number of deaths within 30-day of hospital admission in pneumonia patients, multiplied by the national unadjusted 30-day mortality rate. The predicted deaths are adjusted for patient and facility characteristics, while the expected deaths are adjusted for patient characteristics only. |
| Inpatient Medicine | Patient’s overall rating of hospital stay | Patient rating of hospital data is extracted from the SHEP Inpatient survey, basing on the HCAHPS survey to measure patients’ perspectives on hospital care. The CAHPS Hospital Survey is endorsed by the National Quality Forum, an organization established to standardize health care quality measurement and reporting, in 2005. The SAIL Value Model uses a question measuring overall patient satisfaction with hospital care: “Using any number from 0 to 10, where 0 is the worst hospital possible and 10 is the best hospital possible, what number would you use to rate this hospital during your stay?” A facility’s HCAHPS score is measured as the percentage of patients responded 9 and 10. A higher HCAHPS score is preferred than a lower score |
| Inpatient Medicine | Composite inpatient performance (ORYX) | ORYX is an initiative introduced by the Joint Commission in 1997 that integrates patient outcomes of inpatient care and other performance measure data into the accreditation process. Organizations seeking accreditation are required to submit ORYX performance measure data to The Joint Commission. Starting FY15Q4, the ORYX Performance Measure is a combined composite (ORYX90_1) that averages two inpatient VHA composite measure scores: Global Measures (gm90) and Hospital Based Inpatient Psychiatric Services (hbips90). Each composite (gm90 and hbips90) is calculated using the opportunities model which assumes that each Veteran needs and has the opportunity to receive one or more processes of care, but not all Veterans need the same care. This model summarizes the proportion of appropriate care that is delivered. The Global domain contributes 75% and HBIPS 25% weight to the ORYX90_1 score. At facilities where both service lines/patient populations are not represented, the available composite accounts for all the weight for ORYX90_1. Higher ORYX90_1 scores are preferable to lower scores. |
| Inpatient Medicine | Efficiency | For each medical center, clinical and administrative cost efficiency is measured by using stochastic frontier analysis (SFA). SFA is a well-validated approach in assessing operational efficiency with quality of care taken into account. The modeling principle is to estimate the optimal cost (given quality of care) after controlling for risks or confounding factors such as hospital characteristics, and separating random factors that are not under managers’ control from true managerial inefficiency. Based on the optimal cost, an efficiency score is derived for each facility; an efficiency score of 1.00 is most efficient, and values greater than 1.00 are associated with increasing inefficiency. SFA efficiency measurement is a macro model that does not provide ‘actionable’ information for VISNs and facilities to use. The Efficiency Opportunity Grid (EOG) includes numerous models that correlate with SFA efficiency thereby providing ‘actionable’ areas for VISNs and facilities to target. |
| Outpatient Specialty Care | Timeliness in getting specialty care urgent care and routine care appointments (SC Access composite) | Data for these measures are collected through Specialty Care survey. Administration of Specialty care survey began in May 2015. The FY 2015 survey instrument was based on CAHPS Clinician and Group (CG) Visit survey (2.0) which asks about patients’ experience during their recent visit. Beginning FY2016, the specialty care survey was modified to CAHPS CG version 3.0 which assess patients’ experiences in the last 6 months. The specific questions asked patients to rate their experience with getting timely appointments, care and information on a scale of Never, Sometimes, Usually, and Always (questions 6, 8, 10). A facility’s item score is calculated as the percentage of responses that fall in the top category (Always). The Access composite score is calculated as the average of the facility’s scores on the items. A higher value is preferable to a lower value. |
| Outpatient Specialty Care | Patient rating of specialty care providers | Administration of Specialty care survey began in May 2015. The FY2015 survey instrument was based on CAHPS Clinician and Group (CG) Visit survey (2.0) which asks about patients’ experience during their recent visit. Beginning FY2016, the specialty care survey was modified to CAHPS CG version 3.0 which assess patients’ experiences in the last 6 months. Results are aggregated for all specialty care clinics. Patients are asked to rate their specialty care provider on a scale from 0 to 10, with 0 as the worst provider possible and 10 the best provider possible (question 21). A facility’s score is calculated as the percentage of responses that fall in the top two categories (9, 10). A higher value is preferable to a lower value. |
| Primary Care | Ambulatory care sensitive condition (ACSC) hospitalizations | Hospitalizations due to ambulatory care-sensitive conditions (ACSCs) such as hypertension and pneumonia are preventable if ambulatory care is provided in a timely and effective manner. Studies show that effective primary care is associated with fewer ACSC-related hospitalizations. For this reason, the rate of ACSC hospitalizations is accepted as an indicator of access and quality of primary care. ACSC hospitalizations reported on SAIL is a risk adjusted rate of ACSC hospitalizations per 1000 patients, measured as the Observed to Expected ratio (actual number of hospitalizations due to ACSC divided by the predicted number of hospitalizations due to ACSC) multiplied by the VA national rate per 1000 patients. Lower values are preferable to higher values. |
| Primary Care | Days waited for an appointment for urgent care (PCMH Survey) | Data for these measures are collected through the Consumer Assessment of Healthcare Providers and Systems Survey for Patient-Centered Medical Homes (CAHPS PCMH) survey. The survey instrument was developed by the National Committee for Quality Assurance (NCQA) and the CAHPS Consortium, which was sponsored by the Agency for Healthcare Research and Quality (AHRQ), to capture consumer and patient perspectives on healthcare quality. This report focuses on key measures of the patient-centered medical homes experience including access to care, communications with providers, office staff support, comprehensiveness of adult behavioral services, support provided for self-management, discussion of medication decisions, patient's overall rating of the provider, and follow-up on test results. Patients are asked the number of days they waited for an appointment for urgent care (question 7) on a scale of Same Day, 1 Day, 2 to 3 Days, 4 to 7 Days, and More Than 7 Days. A facility’s item score is calculated as the percentage of responses that fall in the top two categories (Same Day, 1 Day). Higher values are preferable to lower values. |
| Primary Care | Timeliness in getting appointments, care and information (PCMH Access composite) | Data for these measures are collected through the Consumer Assessment of Healthcare Providers and Systems Survey for Patient-Centered Medical Homes (CAHPS PCMH) survey. The survey instrument was developed by the National Committee for Quality Assurance (NCQA) and the CAHPS Consortium, which was sponsored by the Agency for Healthcare Research and Quality (AHRQ), to capture consumer and patient perspectives on healthcare quality. This report focuses on key measures of the patient-centered medical homes experience including access to care, communications with providers, office staff support, comprehensiveness of adult behavioral services, support provided for self-management, discussion of medication decisions, patient's overall rating of the provider, and follow-up on test results. Patients are asked to rate their experience with getting timely appointments, care and information on a scale of Never, Sometimes, Usually, and Always (questions 6, 9, 14). A facility’s item score is calculated as the percentage of responses that fall in the top category (Always). The Access composite score is calculated as the average of the facility’s scores on the items. A higher value is preferable to a lower value. |
| Primary Care | Composite outpatient performance | HEDIS is a widely used set of outpatient performance measures. The measures allow consumers to compare health plan performance to other plans and to national or regional benchmarks. HEDIS is one component of the National Committee for Quality Assurance’s (NCQA) accreditation process. The Centers for Medicare and Medicaid Services (CMS) requires hospitals and health systems (e.g., HMOs) to submit HEDIS data and report the measures in the CMS Hospital Compare. The HEDIS-like Performance Measures include two Combined Composites: HED90_1 and HED90_ec. HED90_1 averages three outpatient sample-based VHA Chart Abstracted External Peer Review Program (EPRP) composite measure scores: Behavioral Health Screening (bh90), Prevention (prv90_1) and Tobacco (smg90). Each composite (bh90, prv90_1, smg90) is calculated as the weighted numerator divided by the weighted denominator established per sampling technique. Each composite contributes equally (33.3%) to the HED90_1 Combined Composite Score because they are considered equally important to quality care. HED90_ec averages two population based outpatient Electronic Quality Measure (eQMs) composite measure scores: Diabetes (dmg90_ec) and Ischemic Heart (ihd90_ec). Each composite contributes equally (50%) to the HED90_ec Combined Composite Score because they are considered equally important to quality care. Composite scores, which are only reported quarterly and annually, are calculated using a modified version of the opportunities model to capture as much information as possible. The model summarizes the proportion of appropriate care that is delivered over a certain period. The denominator is the sum of opportunities (across the population of Veterans) to receive appropriate care across a set of individual measures. The numerator is the sum of the components of appropriate care that are delivered. Higher HED90_1 and HED90_ec scores are preferable to lower scores. |
| Primary care | Patient rating of primary care providers | Data for this measure is collected through the Consumer Assessment of Healthcare Providers and Systems Survey for Patient-Centered Medical Homes (CAHPS PCMH) survey. The survey instrument was developed by the National Committee for Quality Assurance (NCQA) and the CAHPS Consortium, which was sponsored by the Agency for Healthcare Research and Quality (AHRQ), to capture consumer and patient perspectives on healthcare quality. This report focuses on key measures of the patient-centered medical homes experience including access to care, communications with providers, office staff support, comprehensiveness of adult behavioral services, support provided for self-management, discussion of medication decisions, patient's overall rating of the provider, and follow-up on test results. Patients are asked to rate their primary care provider on a scale from 0 to 10, with 0 as the worst provider possible and 10 the best provider possible (question 32). A facility’s score is calculated as the percentage of responses that fall in the top two categories (9, 10). A higher value is preferable to a lower value. |
| Human Resources | Employee rating - best places to work  (includes separate measures of overall job satisfaction, satisfaction with organization, and organizational commitment) | The Best Places to Work score is an annual ranking of U.S. government agencies using data from the Federal Employee Viewpoint Survey (FEVS). It is a composite score ranging from 0-100 points and is calculated by the Partnership for Public Service. In 2013, the AES started collecting the same Best Places to Work survey measures used in the FEVS. These AES measures are then calculated by the Partnership for Public Service to derive Best Places to Work scores across VA at network/area, facility/office, and workgroup levels. Both FEVS and AES Best Places to work scores are measured using three items with the calculation based on the weighted percentage of positive responses (0-100%) of “Very Satisfied/Satisfied” or “Strongly Agree/Agree” to:  • Overall Satisfaction: Considering everything, how satisfied are you with your job?  • Organization Satisfaction: Considering everything, how satisfied are you with your organization?  • Organizational Commitment: I recommend my organization as a good place to work.  Higher scores are preferable to lower scores. |
| Human Resources | Registered nurse turnover rate | The registered nurse (RN) turnover rate measures losses of RNs, a key indicator in Magnet Journey, Joint Commission performance monitor, as well as other national and local reports. SAIL measures losses due to termination and quit (voluntary separation), excluding turnover due to retirement. This measure is limited to registered nurses who are Pay Permanent staff, excluding Nurse Practitioners, Clinical Nurse Specialists, students, fellows and trainees. Facility quit rate (regrettable losses): Voluntary resignations and transfers out of the selected Facility. This turnover rate is especially important to analyze since these losses are voluntary and potentially preventable. Termination rate: Terminations including resignations and retirements in lieu of termination, but excluding losses to military, transfers and expired appointments. A lower turnover rate is preferable to a higher rate. |

*Measure definitions from the U.S. Department of Veteran Affairs Strategic Analytics for Improvement and Learning (SAIL) Value Model; <https://www.va.gov/QUALITYOFCARE/measure-up/SAIL_definitions.asp>.
